# Prevalence of Clinical Periodontitis and Putative Periodontal Pathogens among South Indian Pregnant Women

**DOI:** 10.1155/2014/420149

**Published:** 2014-05-12

**Authors:** Chaitanya Tellapragada, Vandana Kalwaje Eshwara, Shashidhar Acharya, Parvati Bhat, Asha Kamath, Shashidhar Vishwanath, Chiranjay Mukhopadhyay

**Affiliations:** ^1^Department of Microbiology, Kasturba Medical College, Manipal University, Manipal, Karnataka 576104, India; ^2^Department of Community Dentistry, Manipal College of Dental Sciences, Manipal University, Manipal, Karnataka 576104, India; ^3^Department of Obstetrics and Gynecology, Melaka Manipal Medical College, Manipal University, Manipal, Karnataka 576104, India; ^4^Department of Community Medicine, Kasturba Medical College, Manipal University, Manipal, Karnataka 576104, India

## Abstract

In view of recent understanding of the association of periodontal infections and adverse pregnancy outcomes, the present investigation was undertaken to study the periodontal infections among 390 asymptomatic pregnant women and to find an association of bacterial etiologies with the disease. Prevalence of gingivitis was 38% and clinical periodontitis was 10% among the study population. Subgingival plaque specimens were subjected to multiplex PCR targeting ten putative periodontopathogenic bacteria. Among the periodontitis group, high detection rates of *Porphyromonas gingivalis* (56%), *Prevotella nigrescens* (44%), *Treponema denticola* (32%), and *Prevotella intermedius* (24%) were noted along with significant association with the disease (*P* < 0.05).

## 1. Introduction

The causal relation of anaerobic Gram-negative bacilli and periodontal infections is well documented. Bidirectional relationship of periodontal and several systemic illnesses is increasingly being reported. Among these, influence of periodontal infections on adverse pregnancy outcomes is largely emphasized [1–3]. Among more than 700 bacterial species present in the human oral cavity, presence of one or more bacteria belonging to “red complex” has been associated with deep pockets and periodontal infections [4]. Ten putative periodontal pathogens, namely,* Porphyromonas gingivalis* (Pg),* Tannerella forsythia *(Tf),* Prevotella intermedia* (Pi),* Prevotella nigrescens* (Pn),* Eikenella corrodens* (Ec),* Campylobacter rectus *(Cr),* Capnocytophaga ochracea *(Co),* Capnocytophaga sputigens *(Cs),* Aggregatibacter actinomycetemcomitans* (Aa), and* Treponema denticola* (Td) have been studied among various age and ethnic groups to find their association with periodontal infections [5, 6]. Globally, there have been estimates of 40–60% women experiencing gingivitis during their pregnancy [1]. However, paucity of data about the prevalence of periodontitis and the etiological agents from asymptomatic Indian pregnant women is quite evident. Here, we present a study to estimate the prevalence of periodontal infections among asymptomatic pregnant women along with finding an association of microbiological etiologies with the disease.

## 2. Material and Methods

### 2.1. Study Design

This was a cross-sectional hospital-based study to estimate the prevalence of periodontal infections among a cohort of antenatal women and find the associated microbial etiologies.

### 2.2. Study Population

A total of 390 pregnant women of 18–35 years of age within 8–24 weeks of gestation attending antenatal clinic at the Department of Obstetrics and Gynecology at Dr. TMA. Pai Hospital, Udupi, Karnataka, India, during a time period of one year (July 2012–June 2013) were included in the study. The study was approved by the Institutional Ethics Committee. Women who consented to participate in the study with no history of smoking and alcohol consumption were recruited for the study.

### 2.3. Periodontal Examination

Detailed periodontal examinations for assessing the clinical parameters like calculus index (CI), gingival index (GI), plaque index (PI), community periodontal indices (CPI), and loss of attachment (LOA) were carried out for all the women as described previously [7]. CI, PI, and GI were used to examine the dental calculus, plaque, and gingival bleeding, respectively, in all the women. They were measured by probing on the tooth surfaces of 6 index teeth in the oral cavity. Individual index scores were calculated by summing of the scores divided by the number of teeth examined. Based on the GI scores obtained, study subjects were stratified as those having mild, moderate, and severe forms of gingivitis. CPI was used for assessing clinical periodontitis, which had two components, namely, CPI and LOA. CPI scoring range consisted of healthy tissue (no signs of disease) to pathological periodontal pocket (6 mm or more). On clinical examination, women with a pathological periodontal pocket depth of >4 mm, diagnostic of periodontitis, were further examined to measure the loss of attachment levels. LOA scores ranged from minimal loss of attachment (0–3 mm) to an extensive loss of attachment (12 mm or more).

### 2.4. Microbiological Examination

Subgingival plaque samples collected during the periodontal examination were transported in sterile phosphate buffered saline to the laboratory for microbiological examination. Two mL of the specimen was thoroughly vortexed to homogenize the plaque and then centrifuged to pellet. DNA extraction from the pellet was done using QiaAMP DNA mini kit (Qiagen, Hilden, Germany) according to the manufacturer's instructions. The extracted DNA was then subjected to multiplex PCR in two batches (PCR I and PCR II) using species specific primers (Table 1) targeting* P. gingivalis*,* P. intermedius*,* P. nigrescens*,* T. forsythia*,* A. actinomycetemcomitans*,* C. rectus*,* C. ochracea*,* C. sputigens*,* E. corrodens*, and* T. denticola* [5]. The PCR reaction mixture for each sample comprised of 15 *μ*L of PCR Ready Mix (Genei Pvt. Ltd., Bangalore, India), 5 *μ*L of deionized molecular grade water (HiMedia. Pvt. Ltd., Mumbai, India), and 10 picomol of each primer. The amplification conditions for both PCR I and PCR II were similar except for a difference in the annealing temperatures. The PCR was performed using Eppendorf Mastercycler Gradient (Eppendorf, Hamburg, Germany) and the amplification cycles were comprised of initial denaturation of 95°C for 5 min, 35 cycles of 95°C for 30 sec, 60°C for 30 sec (PCR I), 55°C for 30 sec (PCR II), and 72°C for 1 minute followed by a final extension of 72°C for 7 mins. The amplicons were then visualized using 2% agarose incorporated with 1% ethidium bromide (Figure 5) under UV transilluminator (Vilber Lourmat SAS, Marne-la-Vallée, France).

### 2.5. Statistical Analysis

Statistical analysis was performed using SPSS 16 (ver. 16.0, IL, USA). Prevalence of disease and bacterial etiologies were analyzed using descriptive statistical tools. Chi square and logistic regression models were used to study the association between etiological agents and the disease.

## 3. Results

**Figure 1:**
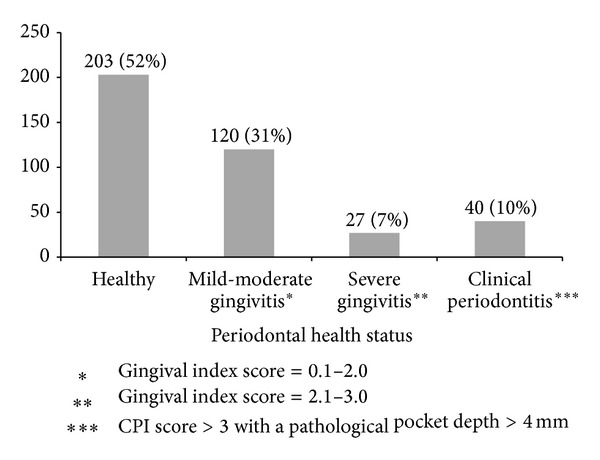
Periodontal health status based on CPI scoring system among study population.

**Figure 2:**
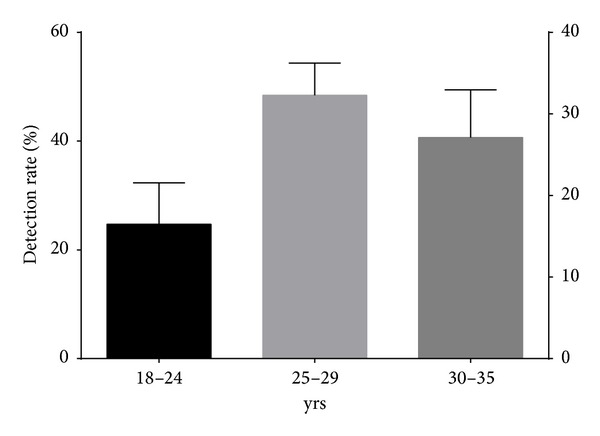
Mean detection rates of periodontal pathogens among women of various age groups.

**Figure 3:**
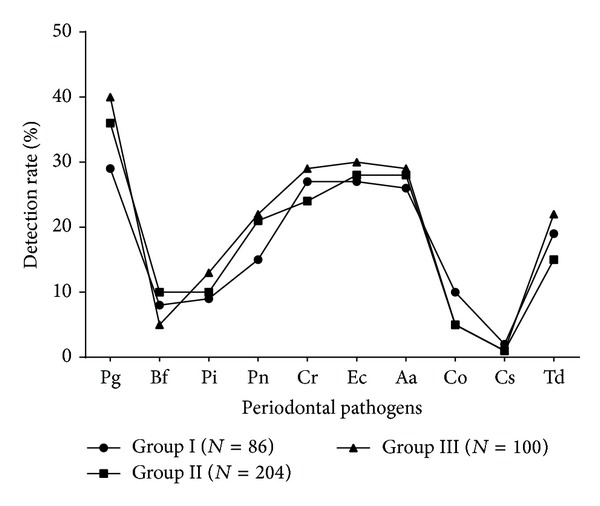
Detection rate of periodontal pathogens among various age groups.

**Figure 4:**
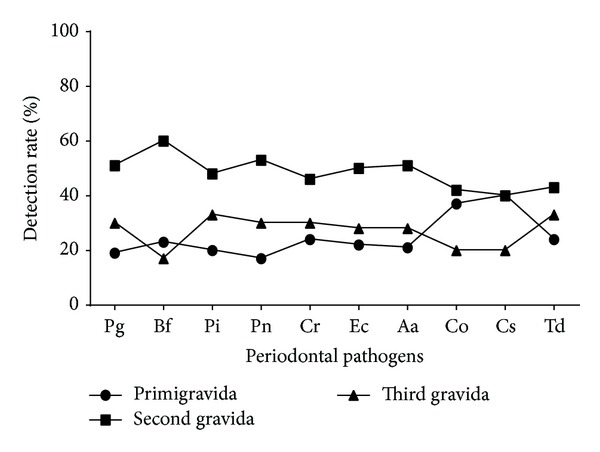
Detection rate of periodontal pathogens among women with varying parity.

**Figure 5:**
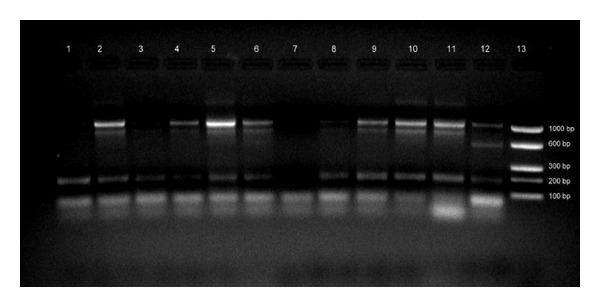
Agarose gel electrophoresis of amplicons produced by PCR II lanes 1, 3, and 8 (Cs); lanes 2, 4, 5, 6, 8, 9, 10, and 11 (Cs and Pn); lane 12 (Cs and Pi); and lane 7 (negative control).

The mean age of study population was 26 years (SD ± 3.4 yrs). Mean gestational period during sampling and periodontal examination was 18 weeks (SD ± 3.5 weeks). Of the 390 women tested, 40 (10%) and 147 (38%) were diagnosed with clinical periodontitis and gingivitis, respectively, as per the community periodontal index (CPI) scoring system (Figure 1). the most frequently detected bacterial agents among the study population were* P. gingivalis* (*n* = 139; 36%),* E. corrodens *(*n* = 110; 28%),* A. actinomycetemcomitans *(*n* = 109; 28%), and* C. rectus *(*n* = 103; 26%) (Table 2). Among the women diagnosed with clinical periodontitis, detection of Pg, Pi, Pn, and Td was more common with a significant association (*P* < 0.05) on Chi square and univariate logistic regression analysis (Table 2).* C. rectus* had a significant association with gingivitis along with* P. gingivalis*,* P. intermedius*,* P. nigrescens*, and* T. denticola* in our study population. Detection rate of* A. actinomycetemcomitans* was noteworthy in periodontitis (16/40, 40%) although statistically significant association was not observed (*P* = 0.08). Maximum detection rates for individual pathogens and their mean detection rate were found in Group II (25–29 yrs) subjects, followed by Group III (30–35 yrs), and least among Group I (18–24 yrs), though the difference among groups was not statistically significant (*P* = 0.72) (Figures 2 and 3). Maximum detection rates of periodontal pathogens were seen among second gravida women followed by third gravida and the least among primigravida women, though statistically significant difference among the groups was not observed (*P* > 0.05) for all the periodontal pathogens tested (Figure 4). Associations of baseline socioeconomic and demographic characteristics of our study population with clinical periodontitis are depicted in Table 3.

## 4. Discussion

Woman's risk of periodontal disease increases during pregnancy under the influence of maternal hormones. Studies indicate that periodontal infection can lead to placental-fetal exposure and, when coupled with a fetal inflammatory response, can lead to preterm delivery [8]. Among several subgingival bacterial agents, few members drew attention towards dual implications in periodontal infections and adverse pregnancy outcomes. While there is substantial evidence indicating association of “red complexes” with severe periodontal infections, data is lacking on etiological analysis of periodontal pathogens in pregnant population [4]. In the present study, we explored the etiologies of periodontal infections in a cohort of pregnant women attending routine antenatal checkup. Prevalence rates of gingivitis and periodontitis among pregnant women vary according to the age, ethnicity, socioeconomic state, and life style habits [9–11]. From our study, we noticed higher rates of clinical periodontitis and periodontal pathogen detection among women within the age group of (25–29 yrs). However, our study population was more homogenous in terms of life style habits, socioeconomic status, and ethnicity compared to previous reports [10]. Also, we noticed a positive association of level of education of pregnant women and their periodontal health status (*P* = 0.029) which is similar to the findings reported previously [12]. The rate of periodontitis, in our study, is slightly lower than that reported elsewhere probably due to study population comprising women of lower age group from middle and upper middle income category and nonsmokers [10, 12]. Many researchers reported higher detection rates of* P. gingivalis*,* T. forsythia*,* P. intermedius*, and* T. denticola* among patients with periodontitis [4, 13]. Similar findings were observed in our study with a significant detection rate and association of* P. gingivalis*,* P. intermedius*,* P. nigrescens*, and* T. denticola* among women with periodontitis. This finding reemphasizes their role as primary periodontopathogenic bacteria. These bacteria along with* C. rectus* are known to evoke potent inflammatory responses in pregnant women that can lead to adverse pregnancy outcomes like preterm birth and low birth weight [14]. Higher detection rates of “orange complex” members (*P. intermedius*,* P. nigrescens*, and* C. rectus*) in our study along with* P. gingivalis* and* T. denticola* (red complex) indicate a coexistence of orange and red complex members among diseased as compared to the healthy group [4]. Previously reported pathogens like* T. forsythia*,* E. corrodens*,* A. actinomycetemcomitans*,* C. ochracea*, and* C. sputigens* had no statistically significant association (*P* > 0.05) either with gingivitis or with periodontitis in the present study population. Detection of these bacteria can be a geographical phenomenon and might depend on the depth of the pathological pocket at which the samples were collected [15]. From our study, we could determine the magnitude of gingivitis and periodontitis among asymptomatic South Indian pregnant women and find the associated etiological agents. Our findings of* P. nigrescens* as an independent risk factor for periodontitis and* C. rectus* for gingivitis suggest the utility of these bacteria as diagnostic markers for periodontal disease screening. Incorporation of routine clinical and microbiological examination for these asymptomatic infections in antenatal care can help in combating the adverse pregnancy outcomes.

## 5. Conclusion

Periodontal diseases of infectious origin are a major concern in pregnant women due to their implications in adverse pregnancy outcomes. Apart from established pathogens associated with periodontal infections, likelihood of new pathogens may not be ruled out. Sensitive molecular techniques are useful in the detection of infection markers of periodontal diseases during the pregnancy.

## Figures and Tables

**Table 1:** Species specific primers used in our study.

Name of bacteria	Forward primer (5′-3′)	Reverse primer (5′-3′)	*T*_a_** (°C)	Amplicon size (bp)
Pg	TGTAGATGACTGATGGTGAAAACC	ACGTCATCCCCACCTTCCTC	60	197
Tf	GCGTATGTAACCTGCCCGCA	TGCTTCAGTGTCAGTTATACCT	60	641
Pi	TTTGTTGGGGAGTAAAGCGGG	TCAACATCTCTGTATCCTGCGT	55	575
Pn	ATGAAACAAAGGTTTTCCGGTAAG	CCCACGTCTCTGTGGGCTGCGA	55	804
Cr	TTTCGGAGCGTAAACTCCTTTTC	TTTCTGCAAGCAGACACTCTT	60	598
Ec	CTAATACCGCATACGTCCTAAG	CTACTAAGCAATCAAGTTGCCC	60	688
Aa	AGAGTTTGATCCTGGCTCAG	CACTTAAAGGTCCGCCTACGTGCC	60*	593
Co	AGAGTTTGATCCTGGCTCAG	GATGCCGTCCCTATATACGGGG	55	185
Cs	AGAGTTTGATCCTGGCTCAG	GATGCCGCTCCTATATACCATTAGG	55*	185
Td	AAGGCGGTAGAGCCGCTCA	AGCCGCTGTCGAAAAGCCCA	55	311

*Confirmation was done using a separate run of PCR to differentiate from other bacteria in the same batch of PCR producing similar size of amplicons.

**T_*a*_: Annealing temperature.

**Table 2:** Detection rates and association with periodontal disease of ten periodontopathogenic bacteria.

Bacterial agent	Overall detection rate (*N* = 390) *n* (%)	Detection in clinical gingivitis (*N* = 147) *n* (%)	Detection in clinical periodontitis (*N* = 40) *n* (%)	*P* value OR (95% CI)
*P*. *gingivalis *	139 (36)	44 (30)	23 (56)	**0.004** 2.6 (1.35–5.15)
*E*. *corrodens *	110 (28)	43 (29)	16 (39)	0.11 1.7 (0.87–3.43)
*A*. *actinomycetemcomitans *	109 (28)	41 (28)	16 (39)	0.08 1.8 (0.92–3.64)
*C*. *rectus *	103 (26)	48 (33)	10 (24)	**0.04** 1.8 (1.01–3.17)
*P*. *nigrescens *	81 (21)	30 (20)	18 (44)	<0.001 3.9 (1.97–7.95)
*T*. *denticola *	73 (19)	22 (15)	13 (32)	**0.01** 2.63 (1.2–5.51)
*P*. *intermedius *	43 (11)	15 (10)	10 (24)	**0.005** 3.2 (1.42–7.46)
*T*. *forsythia *	30 (8)	10 (6)	2 (5)	0.54 0.5 (0.12–2.4)
*C*. *ochraceum *	24 (6)	7 (4)	4 (10)	0.42 1.59 (0.5–4.98)
*C*. *sputigens *	5 (1)	2 (1)	0	—

**Table 3:** Association of baseline sociodemographic characteristics with clinical periodontitis.

Baseline characteristics (*N* = 390)	Healthy group (*N* = 203)	Clinical periodontitis (*N* = 40)	*P* value (*χ* ^2^ Test)
Age			
18–24 yrs	60 (30%)	8 (20%)	0.79
25–29 yrs	97 (48%)	20 (50%)	
30–35 yrs	45 (22%)	12 (30%)	
Level of education			
Primary (up to class VII)	32 (16%)	9 (22%)	0.225
Secondary (up to class X)	73 (36%)	7 (17%)	
Preuniversity college (up to class XII)	45 (22%)	13 (32%)	
Graduate and above	53 (26%)	11 (27%)	
Household monthly income (INR)			
<5000	27 (13%)	2 (5%)	0.134
5001–10,000	59 (29%)	12 (30%)	
10,001–15,000	54 (26%)	12 (30%)	
>15,001	63 (31%)	14 (35%)	
Parity index			
Primigravida	117 (57%)	7 (17%)	0.161
Second gravida	62 (30%)	21 (52%)	
Third gravida	44 (23%)	12 (30%)	
